# How can Community Music Help Address Loneliness in Contexts of Social Marginalisation? Insights From Two Music for Social Connection Programs

**DOI:** 10.1177/2752535X241304084

**Published:** 2024-11-29

**Authors:** Emma Heard, Brydie-Leigh Bartleet, Joel Spence, Kylie Dean, Sam Eyles, Jenny Martinelli, Katie McGuire

**Affiliations:** 1 Creative Arts Research Institute, 5723 Griffith University, Queensland, Australia; 2 Community Plus+ West End Community House, Queensland, Australia; 3 153346 Micah Projects, Queensland, Australia; 4 Independent Artist, Queensland, Australia

**Keywords:** music, loneliness, social connection, community, wellbeing, inequity, inequality

## Abstract

This study explores how participatory music programs can help build social connection for people experiencing loneliness in contexts of social marginalisation. Loneliness is a growing, global public health issue with social and structural drivers. There is an urgent need to investigate innovative approaches to programming that go beyond opportunities for social contact to address the multiple domains of loneliness. Designed collaboratively with two social sector organisations in an urban context in Australia, this study presents outcomes from two community programs that involved groups of people experiencing or at risk of loneliness engaging in music together. Using a qualitative methodology that included semi-structured and brief interviews, focus groups and ethnographic observation, researchers identified positive shifts in relation to social loneliness (related to social contact), emotional loneliness (related to social bonds and meaningful connections), and existential loneliness (related to community connection and acceptance) for participants of the music programs. This study contributes to an urgent gap in understanding effective programming to support people experiencing loneliness and bolsters emerging evidence about the role arts can play in strengthening health, community and social sector efforts to address inequity.

## Introduction

Loneliness has emerged as global public health priority,^1^ with significant implications for the wellbeing of individuals, communities and societies the world over.^2,3^ The World Health Organization estimates that globally one in four older adults are socially isolated and between five and 15 percent of adolescents experience loneliness.^4^ In Australia, one in four people feel lonely at least some of the time and social disconnection is estimated to cost the Australian economy between $2.7 and $6.7 billion dollars annually including through the loss of workplace productivity and increased engagement with healthcare services.^5,6^ Evidence further indicates that people experiencing marginalisation are particularly susceptible to social disconnection including people living in poverty and with low socio-economic status, people with disabilities, people from culturally and linguistically diverse backgrounds, and survivors of domestic and family violence.^3,6,7^ This highlights loneliness as a social justice issue, with social inequity shaping structural forces that influence opportunities and capacity to be connected.^8^

Loneliness is complex and can be experienced socially, emotionally and existentially.^9^ Social loneliness is characterised by feeling isolated and deprived of companionship. This includes a subjective sense of inadequate meaningful connections or discrepancy between desired and actual social interactions and relationships, as well as social isolation which refers to an objective paucity of contact and interaction with others.^9,10^ Emotional loneliness is associated with social loneliness and characterised by feelings of abandonment and exclusion often related to relational issues. Existential loneliness is about a sense of fundamental separateness from others and lack of belonging. Existential loneliness can be felt while in the presence of others and is characterised by feeling like an outsider.^9^ In our understanding, existential loneliness may be particularly relevant for conceptualising loneliness as a social justice issue with marginalisation and disadvantage having the potential to shape experiences of existential loneliness through social exclusion, discrimination and stereotyping. This emerging literature demonstrates that addressing loneliness, and its negative implications for health and wellbeing, requires more than increasing opportunities to be in the company of others, and must support connection across multiple domains.

Globally, programs aiming to address loneliness are increasing, and while some evidence is promising, there is a lack of rigorous, high-quality research to support practice and policy. While many programs are aimed at supporting individuals to be better connected by increasing social contact and enhancing social skills, there have been few attempts to address the structural barriers that underpin loneliness.^8^ There is a key gap in research exploring loneliness with people experiencing social inequity and disadvantage (including understanding the structural barriers they face), and there are calls for increased collaboration across social sectors to support the development, implementation, and evaluation of programs that can work with the complexity of community contexts.^2,8,9,11–13^ Emerging literature is demonstrating that creative programs drawing on the arts have potential to increase feelings of social connection^14,15^ and can work across socio-ecological levels to address structural foundations of inequity.^16–18^ Music programs may hold particular promise for addressing the complex dimensions of loneliness.

### Music for Addressing Loneliness

Within both music and social sector scholarship, the potential for musical engagement to address the growing issue of loneliness across diverse contexts and groups is increasingly recognised. During the COVID-19 pandemic, people used music to cope with stresses, anxieties, and daily challenges associated with isolation.^19,20^ Music can act as a ‘social surrogate’ reducing senses of loneliness and improving mood after social or interpersonal loss.^21^ But the benefits of engaging in music, particularly with others, go beyond coping with social isolation and can support “social bonding and connection.”^22 ^(p.9)^^ A recent meta-ethnography investigating benefits of engaging in music for mental wellbeing found that people can develop connections and strong bonds with others, and find a sense of connection with their wider community.^12^ This literature builds on a solid evidence base demonstrating that participatory music programs can have positive implications for people who are experiencing particular health issues, such as mental illness, cognitive impairments, dementia, and chronic pain, by supporting different modes of communication and fostering a sense of community and connection with others who share similar experiences.^22–28^ Group singing programs in particular are recognised for their ability to foster a sense of community, belonging and inclusion with diverse groups.^28–30^ Community music programs are defined as inclusive, locally-embedded, community-led opportunities for engaging in music.^31^ A growing body of literature is demonstrating that community music programs, may have a unique capacity to affect positive change not only for individuals but also to create opportunities for shifting systemic issues that lie at the heart of social inequity.^18,31–33^ This research offers some insights for investigating the role for music in addressing loneliness as a complex issue experienced across social, emotional and existential domains.^8^

Literature suggests musical engagement may have a role to play in addressing the growing issue of loneliness but key gaps in knowledge remain. Dingle and colleagues^22^ suggest music sharing and song writing show promise for supporting social connection but point to the need for deeper explorations about the types of musical engagement that might work best to address different aspects of wellbeing. Additionally, there is a lack of research exploring programs that address loneliness with people experiencing disadvantage and marginalisation, despite these groups being at increased risk.^3^ Finally, most studies have explored social connection outcomes of musical engagement in the context of wider individual wellbeing; however, there is a dearth of in-depth investigations exploring how music programs might work to specifically address the complexity of loneliness across social, emotional and existential domains, including structural barriers that shape inequitable experiences of loneliness.

The study responds to these gaps, exploring community music programs in the context of addressing loneliness. We investigate social connection outcomes of two community music programs developed in collaboration with community-based social sector organisations and conducted with people experiencing or at risk of loneliness. One program involved attending live music events as a group and one program involved collaborative song writing workshops.

## Research Design

This study used a qualitative research design to understand how people experiencing a range of challenges related to physical and mental health and marginalisation perceive their experience of community music programs in relation to social connection. Social connection can be understood as “the opposite of loneliness” and is an important alternative to deficit approaches.^14(^^p.^^1209)^ It centres on individual agency (what people can do), and resourcefulness to adapt to social circumstances and remain socially engaged.^34,35^ This study takes a social justice approach, understanding social connection as a right that may be shaped by socio-structural forces.^8^

### Research Question

How might community music programs support social connection for people experiencing or at risk of loneliness?

### Researcher Positionality

This study was a collaboration between Griffith University and two community-based social sector organisations working in an inner-city area supporting people experiencing a wide range of challenges related to disadvantage and marginalisation including: homelessness, poverty, domestic and family violence, historic abuse, trauma and mental illness, and disability. The team consisted of four community program facilitators (two from each social sector organisation) and three researchers from Griffith University.

Micah Projects services people facing hardship, adversity and injustice through a range of programs related to homelessness, poverty, domestic and family violence, and community connection. SE formerly led the Creative Community Development team at Micah Projects supporting marginalised participants to actively participate in their community, breaking social isolation and building community. He is a practicing visual artist working in the community where this study was conducted. SE is of Anglo-Celtic heritage. KM is a practicing visual artist and arts facilitator who is currently involved with a creative community arts development and creative engagement programs through Micah Projects. KM has a significant history in designing workshops, providing professional mentorship, and fostering partnerships to promote empowerment and recognition for artists facing social exclusion. KM has Anglo-Celtic heritage.

Community Plus + West End Community House is a neighbourhood centre providing space for people to meet, use facilities, create connections, learn new things, and access support. KD is a proud Gubbi Gubbi woman who has worked in community services for more than 20 years. As a First Nations Community Development Worker with Community Plus + West End Community House, KD has held space for community and mob for a number of years and is passionate about connecting community and social inclusion. JM is an established musician and skilled community music facilitator of First Nations and European heritage. Having been involved with participatory music for wellbeing since childhood, JM was born into community development work and is currently working in homelessness support and is associated with Community Plus + West End Community House as a volunteer running a community music initiative.

Griffith University has a campus located geographically close to the community where this study was conducted. EH is a social and health researcher with experience and interest in arts-based and qualitative research methods. She is of European heritage and has lived in the community where this study was implemented most of her life. BLB is a community music researcher and practitioner who has worked in a wide range of complex contexts from remote Australia to prisons, health equity contexts and war affected cities. She is a first-generation migrant from South Africa and a frequent visitor to the community where this study was implemented. JS is an Australian researcher with European and First Nations heritage. He is a community music facilitator with tertiary qualifications in music education, social work and the arts.

### Community Context

This article reports on outcomes from two community music programs conducted in 2023 in an inner-city, urban area of Meanjin/Brisbane (Queensland, Australia). This area is one of the oldest suburbs of Meanjin/Brisbane, taken from the Turrbal and Jagera/Yuggera people by colonial forces during the (ongoing) invasion of Australia. The last decade has seen growing inequity within the area resulting from rapid gentrification and over development, a rising cost of living crisis, and climate-related disasters including flooding and heatwaves.^36–39^ This has seen rising homelessness and increasing social disconnection for many members of the community, particularly those experiencing marginalisation.^40^

With recognition of the social stigma and barriers to social inclusion and connection that many community members face, the team of authors worked with members of the community to co-design two programs centring on collaborative engagement with music for the purposes of addressing loneliness.^9^

### Two Co-designed Music Programs

With recognition that community contribution is essential to the design and implementation of programs that seek to support health and wellbeing,^41^ two music programs were co-designed in collaboration with the two community-based social sector organisations. Program development was consistent with best practice in community music including non-hierarchical facilitation, active participation of everyone present including facilitators and researchers, shared decision-making with participants, and inclusion and invitation to a wide range of community members.^42^

#### Music Appreciation Program

The Music Appreciation Program (MAP) involved a group of participants from Micah Projects, who were experiencing or at risk of loneliness attending a live music event together once a month across a 6-month period. MAP involved a total of six outings that included: evenings at a local open mic night held at a social enterprise café supported by Micah Projects, a folk music gig held at a local venue, a street music festival, a performance by a professional symphony orchestra, and a musical production developed by the local First Nations community. Events were chosen for their diversity of musical genres, accessibility, and suggestions from participants. All entry costs were covered by Micah Projects, who also arranged transport for anyone wanting to attend and provided refreshments for each session.

**Figure 1.**
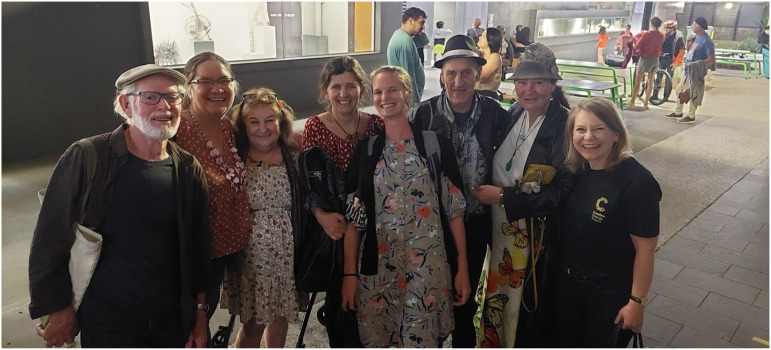
MAP group photo.

Before each session, the group met for snacks and conversation before traveling, via public transport where possible, to the event together. At the end of each session, the group spent time briefly reflecting on the experience together; sometimes this was sitting down to share a non-alcoholic drink and a facilitated discussion, other times it was more informal conversation for example during transport home (Figure 1).

#### Song Writing for Social Connection

**Figure 2.**
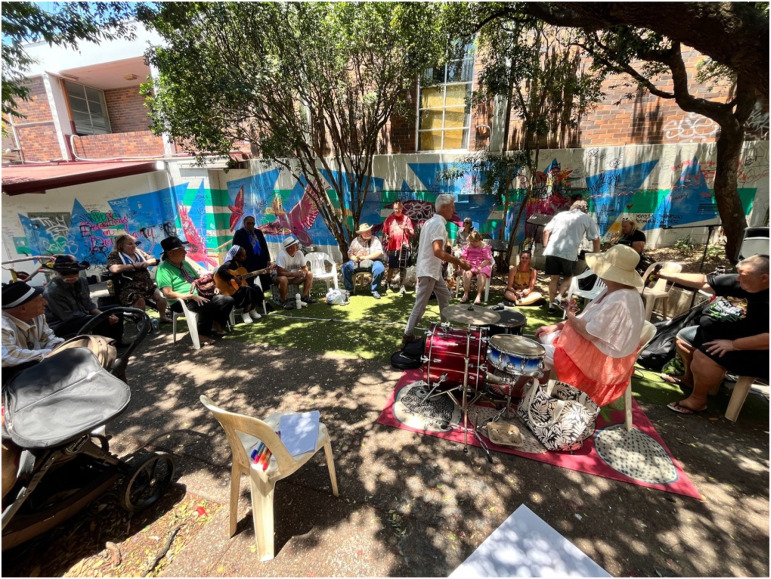
SWSC workshop group.

**Figure 3.**
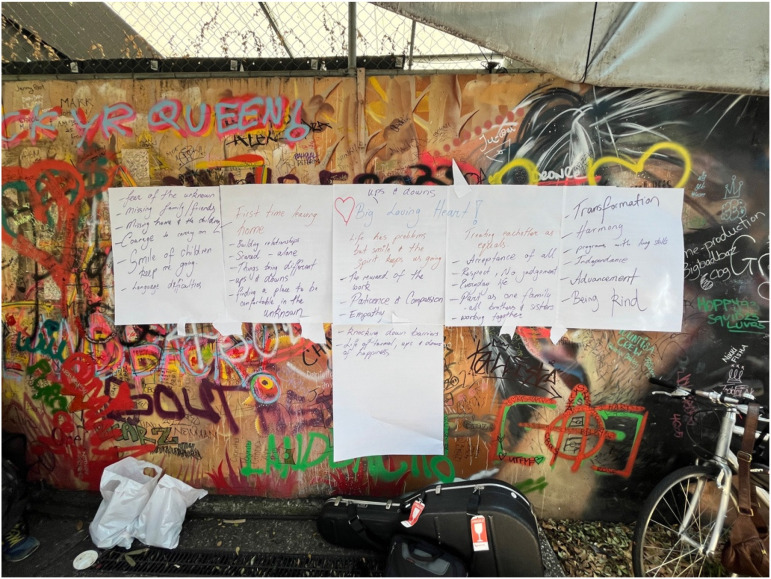
SWSC collaborative lyrics and chord development.

Song Writing for Social Connection (SWSC) consisted of three song writing workshops held fortnightly and conducted with people attached to both partner organisations. The workshops were each 3 hours in length and held in an open, public space where an inclusive community band meet weekly. The sessions were facilitated by the regular facilitator of the community band associated with Community Plus + West End Community House. The sessions were co-facilitated by a singer-song-writer-researcher affiliated Griffith University. One additional researcher and program facilitator from each social sector organisation (all members of the research team) were also present and worked to ensure safety, wellbeing and inclusion. These workshops culminated in the collaborative development of two new songs, written by and for the local community (Figures 2 and 3). Visit tiny.cc/biglovingheart to hear the recording of one of these songs, Big Loving Heart.

## Research Methods

Because we were interested in understanding participants’ experiences and perceptions of the programs in relation to social connection, we approached this research from a constructivist standpoint that recognises reality as socially constructed and contextual.^43^ We wanted to understand the meaning that participants gave to their experiences within the programs and so took a qualitative approach that focused on understanding and centring participants’ experiences in relation to social connection.^44^ Consistent with research methods aligned with constructivist methodologies,^44^ data collection included semi-structured interviews and focus groups with participants before and after the musical engagement activities, as well as researcher observation collected via recorded debrief sessions and field notes. Being conducted in community contexts, participants and data collection methods differed across the two programs, as well as across the individual sessions within each program – these details are provided in the descriptions of data collection across each individual program below.

Ethical clearance was obtained from Griffith University and participants provided informed verbal consent for their data to be collected and used in research outputs, including audio transcriptions of focus groups and interviews, fieldnotes about observations taken by the research team, and photographs taken during the workshops. Specific consent for using images where participants were identifiable was sought by the relevant social sector organisation staff, who confirmed participants were happy for the image to be included in publications (including social media).

### MAP Recruitment and Data Collection

Participants were all community members connected with Micah Projects. Invitations to attend sessions were sent via numerous internal communication channels including email, notice boards at supported social housing, social media, text messages and face-to-face invitations. Each session was attended by between four and 10 people. A researcher and program facilitator from Micah Projects attended each session, participating and guiding the group as necessary. The role of the program facilitator and the researcher was to support inclusion, safety and wellbeing, and also to gather qualitative data through observation and focus group discussions. They also engaged with the music and the discussions as members of this community keen to be socially connected.

A total of 29 individual people attended between one and six of the MAP sessions and participated in audio-recorded pre and post focus groups designed to explore reasons for attending as well as perceived benefits from attending, particularly in relation to social connection. At the end of the program, in-depth interviews were conducted with four participants who had each attended more than three of the events, one had attended all six. These interviews were conducted over the phone, lasted between 15 and 45 min and were audio-recorded. These four participants received a $10 voucher for a local social enterprise café as recognition for their time and input. Focus group and interview questions were developed by the research team with particular guidance from Authors SE, KM, KD and JM who are each deeply embedded within the community and have experience working with participants of each social sector organisation including conducting research and arts-based programs. This was important to ensure questions were accessible and relevant for diverse participants. Questions aimed to capture participants’ perceptions about whether MAP impacted social connection for them, and if so, in what ways. To capture the value of the sessions for participants, questions included ‘What did you like most about this event?’ and ‘What was the most memorable thing about your participation across MAP?’ We used a convenience approach to sampling, whereby all participants who attended the MAP session were invited to take part in the pre and post focus groups and interviews. Focus groups were conducted by the workshop facilitators and interviews were conducted by one of the workshop facilitators and a researcher who was present at all workshops. The team also recorded a debrief session after each event to capture their perceptions and observations about how the events may be benefiting participants and supporting social connection (or not).

### SWSC Recruitment and Data Collection

Invitations to attend the workshops were sent via numerous internal communication channels including email, notice boards at supported social housing, social media and text messages. Being held in an open, public space, community members passing by or who had heard about the workshops through word of mouth also joined. At each session, between 21 and 32 people were present. Being in an open and public space also meant that participants were transient with people coming and going throughout the workshops and contributing at different times. Eighteen ‘core’ attendees actively participated across the 3 hours of one or more workshops. Three of these participants had also attended MAP.

Focus groups with the whole group were conducted at beginning of the first workshop and at the end of the final workshop (lasting between 10 and 20 minutes). Additionally, 10 brief individual interviews were conducted with participants after each workshop (lasting between two and 10 minutes). Sampling was convenience-based, whereby all participants present at the workshops were invited to take part in the pre and post focus groups and the participants who stayed for refreshments after the final workshop were invited to participate in brief interviews. Focus groups and interviews were conducted by the two workshop facilitators and the researcher who were present at the workshops, and audio-recorded. Similarly to MAP, questions were designed with particular input from social sector organisation team members. Questions explored participants’ expectations and hopes for the sessions, as well as perceived outcomes in relation to social connection. Researcher observations about if and how the workshops supported social connection among participants, or not, were captured in recorded debrief sessions.

### Data Analysis

All interviews, focus groups, and team debrief sessions were transcribed verbatim. We adopted a thematic approach to analysis.^45^ One researcher conducted an initial inductive analysis carefully reading and openly coding each transcript to identify outcomes broadly relating to social connection. The research team met as a whole to discuss these initial codes, including reviewing and discussing key quotes, drawing on their diverse professional and lived experiences as artists, community development workers, teachers and social researchers to consider their meaning. Our social justice approach to research and practice, including taking a strengths-based approach, guided decisions as we sorted these initial codes into overarching themes as a team. Authors then considered these themes in relation to literature on loneliness, grouping inductive themes under the three domains of loneliness – social, emotional, and existential, to gain a deeper understanding of the impact of the programs in relation to social connection.^9^ One author then re-read each transcript with a specific focus on each theme under this final framework. To further support rigor and trustworthiness, all members of the research team then reviewed the final coding.

## Findings

Across both programs, a total of 47 individual people participated in the activities. 30 people identified as women and 17 as men, and ages ranged between early twenties and late seventies. Six participants were First Nations, 21 identified as having a disability and 15 participants were living in social or supported housing. Being participants of the partner social sector organisations, most people who participated in the programs were living with significant challenges associated with poverty, (risk and/or history of) homelessness, or trauma and mental health putting them in or at risk of loneliness and/or social exclusion.

To illuminate how musical engagement with others can work to address loneliness, we outline findings across social connection, emotional connection, and existential connection.^9^ We present three themes under each of these categories as outlined in Table 1. Findings from both programs are presented together because insights relevant to all themes were identified in data from both programs. While themes are relevant to both programs, in order to better understand the specific mechanisms affecting social connection, we have identified throughout the Findings when a theme is particularly apparent in data from one program.

**Table 1.** Overview of Findings by Themes.

**Social connection**
Creating unique opportunities to be with others
Creating a supportive environment for connecting with others
Skill sharing and learning with others
**Emotional connection**
Building relationships with others
Creating opportunities for creative self-expression
Connecting people with social support services
**Existential connection**
Connecting and engaging with community
Creating something with others
Contributing and showing care

### Social Connection

Both music programs provided opportunities for developing social connection through creating supportive and accessible opportunities to be with others in ways that allowed people to get to know each other and learn with each other.

#### Creating Unique Opportunities to be with Others

Almost all participants from both programs valued the opportunity to “be with” and engage with others through the music programs. This was particularly pertinent for participants who struggled with social anxiety:

One reason why I'm standing here is because I play computer games and sometimes I play all day and there's not much talking. … I might be lousy with people but I'm good with music. (P18, man, MAP participant)

Relatedly, participants appreciated the opportunity to meet new people in these contexts:

I have met a few nice people. And I'm grateful for it. (P10, woman, MAP and SWSC participant)

For others, the programs helped overcome other challenges related to poor health, mobility issues and poverty, which had limited their ability to socially engage with others. This was particularly salient for participants of MAP, which included transport and supporting the use of public transport, entry costs, refreshments, and holding sessions in central and accessible locations. Similarly, participants of SWSC appreciated the central and accessible location. Program facilitators reflected about how attending music events in the community as a group created opportunities for participants to be able to access these spaces at other times in their lives through familiarisation, including awareness of venues, navigating how to get to them and having a positive experience being there:

[It’s about] normalising those experiences for people that may not have that opportunity. (P20, man, MAP facilitator)

Participants of MAP who lived in social and/or supported housing described how important it was to have these opportunities to socialise with their neighbours and people in the wider community in a safe and positive way:

It was delightful, encouraging, and uplifting. Even the ones that I didn't really like the music of, I thought were still great. I loved it! It was something to get me out of, get us out of, the building and doing something with other people. And it allowed reconnection to meet people, people in the building and other people that are in the music scene, which is great! (P19, woman, MAP participant)

Mutual respect and inclusion were central to the design and facilitation of both programs, and this supported participation:

What it actually does is, that boredom and loneliness disappears with involvement. So feelings of anxieties, of social barriers are broken because once everyone comes in, everyone feels comfortable. Like today … all those anxiety, fear, was all thrown out the window. It was all rolled over the shoulder because we changed into a different zone. And we were in a circle, protected. (P3, man, SWSC participant)

Beyond being an opportunity to be with others, participants articulated how the music specifically supported connection.

#### Creating a Supportive Environment for Connecting with Others

Participants in both programs described how the music had a positive influence on their moods and described their experiences as “uplifting” and “joyful”. This was key for creating an environment where people felt able to interact with others:

Definitely it puts everyone in a light-hearted, kind of happy frame of mind. … it definitely creates a nice vibe and I think that contributes a lot more than what people would consider. (P23, woman, program facilitator)

The music created a supportive environment that facilitated positive engagement with others and minimised social pressure as participants across the programs highlighted how they had something to connect over, an already established common interest and a shared positive experience:

[I]t gives you something to have to focus on ... Like when you catch up, you're thinking about the music, you're talking about the music. Relating through the music, rather than trying to, just blank slate, trying to talk to someone you don't know. … You've already got something in common... (P14, woman, program facilitator)

I think music is a way to connect, it's the connective material. It brings us all together and connects people. (P16, man, SWSC participant)

As a result of mutual respect and safety being embedded throughout the programs’ design and implementation, participants described how the programs supported people to share parts of themselves in safe and inclusive ways. This was important for feeling connected to each other:

I think [it’s] great … because that brings out the whole lives of people, a bit of knowledge and understanding and feeling, you know. (P10, woman, MAP and SWSC participant)

Well, [in other programs] everyone tends to … become like crabs and close up. And when they open up, it's threatening. … [But with this group] there's no fear. All groups don't, can't do that. Many groups separate everyone. There's no cohesion. And this didn't happen. And that was good. (P5, man, MAP and SWSC participant)

This meaningful connection was beneficial for sharing and learning with each other.

#### Skill Sharing and Learning with Others

For many participants across both programs, these safe, supportive environments were conducive to knowledge and skill sharing, and participants described how they valued learning new things from others in the groups:

I'm believing it's a good thing to have something like this because it brings out the best in people. Not only that, people became a bit more communicable and therefore they will bring out some informations good for development and for other people. … Companionship. Sharing information. Yeah, you're gathering information. See we were talking on the way, conversing on the way here and [another participant said] said to me, 'Do you know that Frank Sinatra is already dead?'. I didn't know that. So we are learning from each other. (P24, woman, MAP participant)

[Writing a song together] makes the group that touch more powerful. And communication happened today. … Where everyone's expressing ideas and stuff like that, using their brain. (P3, man, SWSC participant)

The non-hierarchical design of the programs, including the positioning of facilitators and researchers as members of the community and their active engagement in program activities allowed for participants to contribute in different ways and feel comfortable and confident to both share and learn:

People who did not know each other were introduced and shared skills and stories. For example, [one participant] was talking with someone new about learning drums, and [another] was working with [a group of people] on the song intro and chord progression. Many people stayed around for food afterwards and the participants were all chatting and getting to know each other. (P25, woman, program facilitator)

Participants in both programs described how their engagement in the programs encouraged them to pursue broader musical engagement including joining choirs, joint instrument lessons, collaborative song writing and community music groups.

Overall, the programs provided unique, enjoyable and relaxed opportunities for participants to be with and engage with others in meaningful ways including sharing skills and knowledge. Importantly, our findings demonstrate participants in both programs not only experienced an increase in opportunities to connect with others – addressing social loneliness, but these opportunities supported meaningful bonding and relationship building – addressing emotional loneliness.

### Emotional Connection

Beyond providing safe and supportive opportunities to meet and be with other people, the music programs were useful for relationship building, including through moments of self-expression. The programs were vehicles to connect people to both others with similar life experiences and with social support services.

#### Building Relationships with Others

Participants from both programs described “making friends”, developing “companionship” and “camaraderie”, and referred to the groups as “family”. These relationships were built on moments of shared interests and talents through music.

Oh, damn it was really good. Yeah, I thought it was a good chance to show everybody can do something. And share, we all shared something. We shared enthusiams, laughs, friendship, getting together, sharing ideas, you know? I think it's great. I don't know what everybody thinks, I think we should ask everybody else. I'm not the only person. (P10, woman, MAP and SWSC participant)

During the music programs it was common for people to start singing together. For example, during a MAP session a participant would mention a favourite artist and the group would be compelled to sing one of their songs together. Talking about music led to sharing parts of their lives and histories, and this was important for building strong bonds. Participants in both programs stated they would continue to connect with each other outside of the group. MAP participants stated they would consider going to other events together and SWSC participants indicated interest in joining a community band that meets in the same location regularly. Additionally, reconnection and the reestablishment of relationships were facilitated through the programs. The music and sharing that happened around the music helped participants to realise mutual interests and want to spend time together.

I reconnected with [another participant] because we didn't always have a good relationship… [but at the gig] she said, “I know I haven't been very nice to you, but I'd like to be friends”. … I gave her a big hug. Music connects people…. That happened through this [program]. You know, so, fractured relationships were repaired. (P19, woman, MAP participant)

People were swapping phone numbers, sharing music and favourite songs. [Participant C] and [Participant D] seemed to have re-kindled their relationships through the music group, they arrived together and went out to dinner together afterwards. … [Participant E] and [Participant F] shared discussions about music they liked and made plans to sing ‘The Rose’ together at an upcoming open mic night. [Participant C] was very encouraging of [Participant F] joining [a community music group she was a part of]. (P24, woman, program facilitator)

One MAP participant was supported to bring an elderly family member, who was largely immobile, living in residential care and socially isolated, to an event of a professional symphony orchestra. This was particularly significant because the elderly family member had been a member of that orchestra many decades before. The event was an opportunity for the participant to share a mutual interest and have a positive experience with their family member. This opportunity further supported ongoing social connection between the family member and current members of the orchestra.

I printed up a photo collage, really nice photos of [my family member] with people that she knew [from the orchestra]. And so we were able to give her that the other day when we visited her [at the residential care facility]. And so she's got something to remember [the event] by. And then some of the orchestra members came and saw her as well. (P5, man, MAP and SWSC participant)

This bonding was further facilitated through the safe and inclusive opportunities for self-expression created during the programs.

#### Creating Opportunities for Creative Self-Expression

Throughout both programs participants felt comfortable to express themselves through music and this included sharing songs and poems they had written, playing covers of songs that were meaningful for them, and contributing lyrics and ideas for musical arrangements during collaborative song writing sessions. For some this was new or something they did not regularly do. These moments were essential for supporting emotional connection between participants as they facilitated bonding through demonstrating vulnerability and exposing one’s inner self.

[Participant singing a song written for Forgotten Australians.^46^ This participant had contributed to and gave feedback during the writing of this song]

Have you forgotten me? Taken by the government hidden from you.

Innocence stolen from all but the few.

Parted from our family for traits of love denied.

The tyranny of strangers no matter how we cried. Have you forgotten me?

[Two other participants joining in the singing]

Have you forgotten me?

Do you know who I am? Have you forgotten me?

(P27, woman, MAP participant; with P19, man, MAP and SWAC participant and; P14, woman, program facilitator)

As demonstrated by the two other participants joining in the singing above, these gifts of creative self-expression were met with both support and gratitude, and valued as a means through which connection was built.

I feel connected to everybody because everybody had something to give, don't they? And we gave freely. (P2, woman, SWSC participant)

#### Connecting People with Social Support Services

Implementation of both programs in collaboration with multiple social sector organisations was important for supporting emotional connection as it facilitated dialogue between people with similar life experiences and put people in touch with support services.

Whether or not a person attended the session, the invitation was an important mechanism through which to reach people. It gave workers at both social sector organisations an opportunity to demonstrate to a person that they know and care about them as an individual (e.g. that they enjoy music). It was a chance to establish dialogue from a position of strength, rather than to help with a problem. There were numerous examples of this dialogue leading to productive and important conversations about how a participant of the social sector organisation could be better supported.

I message participants and talk to them about what’s coming up. And that's engagement. Otherwise, the only reason you are having contact is because of a problem. It gives you an opportunity to contact people about something positive … (P14, woman, program facilitator)

The inclusive, informal and community contexts within which both the programs took place meant that the programs also reached people who were not currently engaged with support services either due to lack of awareness or distrust associated with institutions. This was achieved by: collaboration across social sector organisations, being in public spaces, open invitations not associated with a particular need or deficit, and mutual respect and participation by all involved.

The overall feelings of the outings I went on, the music, I found that everyone was changed when they came out. I think they were better than when they went in, and I think that is the power of music. … You've got [people] that don't get assistance … they are basically on the fringe, between nothing and whatever they can scrounge, which is a hard road. Probably many of them don't trust anyone. And rightly so. They've been betrayed by the best of them. (P5, man, MAP and SWSC participant)

There were at least two instances where participants were introduced to services being run by the social sector organisations that they were not previously aware of including support services for Forgotten Australians^47^ and connecting First Nations communities.

Each of the above examples demonstrate how the programs offered people opportunities to connect with each other and with service providers in meaningful ways with potential to address emotional loneliness. Our finding further demonstate connection to communities more broadly, with potential to support existential connection.

### Existential Connection

In addition to social and emotional connection, both programs facilitated a sense of existential connection for participants through engagement with the wider community, facilitating the co-creation of something with others, and providing a way to contribute and show care to their community.

#### Connecting and Engaging with Community

For many participants both programs provided opportunities to be in their community in ways that supported a sense of belonging and welcome. Participants described how they had learnt about their community through attending musical performances, including by First Nations musicians, and how this was important for understanding themselves in relation to place and others. Additionally, as demonstrated by the example below, participants had opportunities to be in their community and experience intermittent connection with strangers that could contribute to knowing that people in their community share their culture and values.

[Participant H] told me his story of arriving as a refugee, with nothing, having lost all his belongings in a boat accident on his way. He told me how he played the accordion back in in his home country “before the revolution, before music was banned. Very bad, very bad”. When I asked more about where he was from, he broke into song. A person not connected to our group, at another table, recognised the [traditional] tune. They sang together and spoke across the room for a good while, smiling and laughing. (P25, woman, facilitator)

For participants with disability, the programs were significant as they facilitated a means to be in their community and accepted on their own terms, doing something that was meaningful and enjoyable to them.

And I think one of the other great benefits to something like this is many people with disability don't get the opportunity to go out into community. And if they do, it's not always with who they want to go out with. And it's also not when they want to, or with music. So, it's good to have many people who are marginalised and not necessarily able to get out all the time to go to events such as this. (P22, man, MAP participant)

In addition to engaging with the broader community the programs worked to support existential connection through creating something meaningful with others.

#### Creating Something with Others

During both programs, and SWSC in particular, participants developed a strong sense of connectedness through creating something together, something that they cared about and that represented them.

We're all combined in this song, that we all know from the very seed, when it started off. You know, to build from that first workshop, all of us participating, so we've got a connection to it, and watching how it just flowered out into the song. Wow. We have a personal connection with it. It's not [like when you] play some songs and you've got no idea what the lyrics… mean. This one is voicing our real feelings. (P6, man, SWSC participant)

This was important for facilitating a sense of togetherness that could reduce feelings of alienation or aloneness.

[I]t was a collective thing that everyone wanted to have an input into. So that was definitely, I don't know what the word … would be. But like community and community-wise, yeah. We became a little community within ourselves. (P3, man, SWSC participant)

Additionally, because of connections built during the MAP program, participants were inspired to facilitate a group sing-along at the final program session (an open mic night at a social enterprise café).

#### Contributing and Showing Care

Participants described how both programs, and SWSC in particular, provided opportunities to contribute and demonstrate care for others. For example, one of the collaborative songs written during SWSC was a gift for workers of an orphanage in another country who had shared their story with the group. As one SWSC participant said, “it feels like you’re… contributing.” Another song was an ode to a significant First Nations community worker and an expression of love and gratitude for their contributions. For others, being a part of the groups enabled them to invite others to join and gave them a vehicle to extend friendship and joy to others.

[This person] was walking past me and I thought, ‘Ah there’s a guitar, I'll go tell them about what's happening’. Yep. So I actually pulled that [person] off the street too, the one who is playing the violin today. … She turns out to be hell professional too. Yeah, even better. (P3, man, SWSC participant)

Through opportunities for people to be in the community on their own terms, to create something meaningful together with others, and to contribute to the joy and wellbeing of others, the programs supported existential connection among participants.

## Discussion

This exploratory study demonstrates a potential for community music programs to be effective for supporting social connection for people experiencing or at risk of loneliness, particularly when conducted in community settings and in collaboration with community-based social sector organisations.^48^ Our findings are timely and pertinent given the emerging evidence highlighting loneliness as a global public health concern with significant health and wellbeing implications.^2,3,49^ Our findings highlight that community-led, participatory music programs may be useful for supporting social connection across social, emotional and existential domains of loneliness.

Consistent with emerging evidence, the musical aspects of these programs supported a positive mood among participants and a common interest that facilitated engagement and reduced social anxiety.^14,28,50–52^ This is important given literature pointing to the need to support people who are experiencing loneliness to have positive social interactions, not merely provide opportunities for social contact.^13,53^ Spending time with people does not necessarily reduce the burden of loneliness, and facilitating objective opportunities to be with others is only one aspect of addressing the complexity of loneliness.^53^ Our findings support emerging evidence suggesting that engaging in music with others can facilitate bonding more quickly than other activities, including other arts-based activities, which supports emotional connection.^54^ Participants of both MAP and SWSC reported building relationships with others, and sharing skills and knowledge with others despite the programs only being monthly for 6 months (MAP) and three workshops held fortnightly (SWSC). Recent literature suggests that active engagement with music can have positive influences on the emotional and social aspects of wellbeing^12,55^ and our study bolsters this burgeoning body of literature signifying a role for arts in supporting emotional as well as social connection.^14^

Both MAP and SWSC were accessible in a range of ways that addressed previous practical barriers to social engagement for participants, including transport, refreshments, and no cost, and importantly provided access to spaces and events that participants had not previously felt welcomed in. This introduced the potential for participants to be in these spaces in the future. Being in public and inclusive community spaces allowed for spontaneous and incidental interactions with members of the broader community and these moments of ‘breif social interactions’ can have positive implications for health and wellbeing.^56,57^ Additionally, these opportunities for positive interactions and engagement with the broader community can support a positive sense of place, which is an important element of both social and emotional connection.^9^

Existential loneliness is about a sense of fundamental difference or alienation from others, and these moments of interaction with people in the community have potential to create a sense of belonging and shared values; as demonstrated by the MAP participant with a history of seeking asylum sharing a traditional song from their home country across a courtyard with a stranger. This is consistent with literature suggesting music can foster connection with others through shared culture and heritage, and can play an important role in belonging for refugees and asylum seekers after resettlement.^12,58^ Extending on this, the programs provided opportunities for contribution and showing care, and a chance to create something both meaningful and tangible with others. These outcomes were particularly evident in SWSC where participants highlighted ways in which collaborative song writing allowed them to better understand each other, feel meaningful affiliation with the group and with the broader community, and give something back to their community.^12^ Literature is demonstrating that brief moments of showing care for others, including strangers, can be good for social connection, among other positive health and wellbeing outcomes.^57^

Loneliness is a structural issue as much as a personal one; addressing marginalisation and discrimination at a societal level is essential for combatting loneliness.^8^ It is likely that experiences of emotional and existential loneliness are shaped in part by marginalisation and discrimination. The music programs facilitated people to be in public spaces, including spaces with potential for exclusion and devaluation, safely and on their own terms.^8^ SWSC was held in an open, public space on a main street. It was an opportunity for participants to loudly proclaim a right to belong in that space and to contribute to the shared identity of the wider community.^16,18^ The potential for music and arts programs to address structural foundations of social inequity is underexplored, and this is particularly important given the disproportionate prevalence of loneliness among people experiencing marginalisation.^3,6,7^ Recent literature is exploring the role that the arts and music specifically can play in building social equity.^16,31,32^ This work may provide insights for considering ways to address the structural foundations of loneliness and bolster social connection among disadvantaged and marginalised groups; something that is currently missing from the literature related to loneliness.

Our findings identify some key elements of program design that supported positive social connection outcomes, which can inform planning and implementation within health, community, and social sector organisations. These echo best practice approaches in community music^42,59^ and include non-hierarchical facilitation and active participation, shared decision-making, and authentic inclusion that engenders hospitality within the group and the wider community. The invitation to participate in the music programs was an opportunity for a positive interaction with participants of the community-based social sector organisations, which had constructive implications regardless of whether the person engaged in the music program or not. Further, being implemented in collaboration with two established social sector organisations each supporting people experiencing marginalisation and disadvantage in different ways allowed for participants to be introduced to new support services and to others with similar life experiences. Notably, the success of the programs reported on in this study hinged on the experience and skill of the community facilitators, who each have comprehensive knowledge of both social work and/or community development and participatory arts, as well as deep involvement with the community. These findings are important given recent calls for exploring what makes effective community music facilitators.^60^

### Strengths, Limitations and Future Directions

This study addresses an important gap in current understandings about how creative approaches to social sector programming can be effective for addressing loneliness. The community contexts within which this research was conducted, and the qualitative methods used allowed, participants to engage meaningfully in the data collection processes. This supported authenticity and depth of the data collected, pointing to not only the *what* but illuminating some effective program elements (i.e. the *how*).^12,13^ Future studies could use a deductive approach, and mix-method designs may be useful in particular health and psychology disciplines. Importantly, participants reported enjoying the research aspects of the programs, highlighting how the discussions both strengthened connections with other participants and were empowering in that they provided opportunities to give their perspectives and have their experiences heard and acknowledged.^61^ This process was strengthened by the inclusive and mutually respectful nature of the program design that saw researchers and program facilitators participate and engage with participants during the programs. This may have produced an element of social desirability whereby the participants emphasised favourable views because of their relationships with the facilitators, researchers and each other. Future qualitative research could be strengthened by including data collection methods that allow for deeper investigations considering implications of and for participants’ multiple social locations and intersecting systems of power.^12^ Such investigations are important for social justice research and strengths-based program design.^62,63^

The community contexts meant that participation was not consistent across each program and not all participants’ experiences were captured using our convenience approach to sampling. Additionally, our findings only captured immediate post perspectives and thus, this study provides limited insights into lasting impacts for participants (e.g. if intentions to remain connected with other participants were realised). Longer follow-up would allow for the investigation of lasting impacts and could capture actual behaviour not only intention.

## Conclusion

This study outlines positive outcomes in relation to social connection for participants of two community music programs conducted in an urban context in Australia. These outcomes are significant given the emerging evidence demonstrating loneliness as a global public health priority and the urgent need for programming and policy that goes beyond creating opportunities for social contact to building meaningful social connection and addressing the structural foundations of loneliness.^1,8^ Outcomes from this study suggest community music programs that are designed in collaboration with social sector organisations and grounded in mutual respect, inclusion and hospitality can work to address loneliness across multiple dimensions including strengthening social, emotional and existential connection. Further investigations into the role of participatory music, and arts more broadly, in health, community and social sector programming and policy for addressing loneliness are warranted.
